# Baseline demographic, clinical and multimodal imaging features of young patients with type 2 macular telangiectasia

**DOI:** 10.1186/s40942-023-00485-6

**Published:** 2023-08-09

**Authors:** Nikitha Gurram Reddy, Vishma Prabhu, Sumanth Vinayak Sharma, Isha Acharya, Rubble Mangla, Naresh Kumar Yadav, Jay Chhablani, Raja Narayanan, Ramesh Venkatesh

**Affiliations:** 1 Anand Bajaj Retina Institute, L V Prasad Eye Institute, Kallam Anji Reddy Campus, Hyderabad, 500034 India; 2 Dept. of Retina and Vitreous, Narayana Nethralaya, #121/C, 1st R Block, Chord Road, Rajaji Nagar, Bengaluru, Karnataka 560010 India; 3 Medical Retina and Vitreoretinal Surgery, University of Pittsburgh School of Medicine, 203 Lothrop Street, Suite 800, Pittsburgh, PA 15213 USA; 4 Anand Bajaj Retina Institute, L V Prasad Eye Institute, Indian Health Outcomes, Public Health and Economics Research Centre (IHOPE), Kallam Anji Reddy Campus, Hyderabad, 500034 India

**Keywords:** Macular telangiectasia, Young age, Optical coherence tomography, Visual acuity, Stages

## Abstract

**Purpose:**

Macular telangiectasia (MacTel) type 2 is observed in patients in their 5th–8th decades of life. The clinical and imaging findings in younger patients is unknown in larger cohorts. The study purpose is to report prevalence, baseline clinical and spectral domain optical coherence tomography (SDOCT) findings in young MacTel patients below 40 years.

**Methods:**

This hospital-based, multicentre, retrospective, cross-sectional study included patients between 2011 and 2023. Retinal photographs from multiple imaging techniques were evaluated to diagnose and stage type 2 MacTel and describe their SDOCT findings. Imaging characteristics were correlated with clinical stages and visual acuity.

**Results:**

Among all MacTel patients seen in hospital, prevalence of young MacTel cases less than age 40 was 1.77% (32/1806; 62 eyes). Youngest participant was 34 years, while mean age was 38.44 ± 1.795 years. Sixteen patients (50%) were diabetics. Perifoveal greying (n = 56, 90%) and perifoveal hyperreflective middle retinal layers (n = 47, 76%) were the most prevalent clinical and SDOCT imaging finding respectively. Less than 10% (n = 6) eyes had proliferative disease. Presence of retinal pigment clumps (RPC) (7% vs. 67%; p = 0.002) coincided with proliferative MacTel. Poor vision was associated with presence of outer retinal layer SDOCT findings like outward bending of inner retinal layers (p = 0.047), RPC (p = 0.007), subfoveal neurosensory detachment (p = 0.048) and subretinal neovascular membrane (p = 0.001).

**Conclusion:**

Type 2 MacTel before age 40 is rare, common in women and diabetics, and affects vision in advanced stage. Disease symmetry, comparison with older cases, and longitudinal SDOCT changes in such patients require further study.

## Introduction

Type 2 macular telangiectasia (MacTel) is commonly observed in the older population, primarily impacting individuals between the ages of 40 and 80 years [1]. According to the Beaver Dam Study, a population-based study conducted in the United States, the estimated prevalence of type 2 (MacTel) among individuals aged 43 to 86 years was approximately 0.1% [2]. The diagnosis of type 2 MacTel is typically delayed until patients seek medical attention at an outpatient clinic due to the manifestation of visual symptoms. Typically, cases of type 2 MacTel in the early stages exhibit no apparent symptoms. Hence, the occurrence of type 2 MacTel within younger age cohorts, specifically prior to the onset of the fifth decade, is infrequently documented in scientific papers. However, a limited number of anecdotal reports exist regarding the early presentation of type 2 MacTel and its association with visual complaints [3, 4]. The medical literature documents the occurrence of type 2 MacTel in a case involving an 11-year-old female patient, representing the youngest reported instance of this condition [5]. The utilization of contemporary retinal imaging techniques has the potential to enable the early detection of type 2 MacTel before the manifestation of observable fundus clinical findings. Based on the available information, a comprehensive database of type 2 MacTel cases detailing the clinical and imaging characteristics in individuals under the age of 40 appears to be lacking.

To address this research question, a study was conducted with the aim of describing the prevalence, baseline clinical, and imaging characteristics of a cohort of hospital-based patients with type 2 MacTel who were diagnosed before the age of forty years. The findings of this study have the potential to be a valuable asset for enhancing comprehension of disease pathogenesis, pinpointing the modifiable risk factors accountable for disease onset and prevention, and formulating treatment strategies that could delay the progression to an advanced disease stage, thereby mitigating the risk of vision impairment.

## Methods

In this multicentre, retrospective observational hospital-based study, we identified and reviewed the electronic medical records [eye SMART EMR (Hyderabad, India) and NETRAM Ophthalmology EMR (Delhi, India)] and spectral domain optical coherence tomography (SDOCT) images of patients ≤ 40 years of age who were initially diagnosed with type 2 MacTel between January 2011 to April 2023. The data was collected and compiled from two tertiary eye care institutes in South India. The study adhered with the tenets of the Declaration of Helsinki and was approved by the local Institutional Review Board/Ethics Committee.

The diagnosis of type 2 MacTel was made based on a constellation of clinical findings as described by Gass and Blodi [6] in either one or both eyes and other multimodal imaging features such as perifoveal hyperreflectance on confocal blue reflectance imaging, perifoveal hyperfluorescence in late stages of fundus fluorescein angiography or SDOCT imaging findings as described further. Patients with other concurrent macular pathologies were excluded. Patients with an uncertain type 2 MacTel diagnosis based on clinical examination and on different imaging methods were also excluded. Patients with media opacities that prevented the acquisition of high-quality SDOCT scans were excluded. At the baseline visit, demographic information, Snellen’s best corrected distance visual acuity, retinal examination findings, and SDOCT imaging characteristics were recorded. Perifoveal greying, superficial retinal crystals, right-angled vessels, retinal pigment epithelial plaques, and the presence of subretinal neovascular membrane (SRNVM) were looked for in the macula of these type 2 MacTel eyes.

**Fig. 1:**
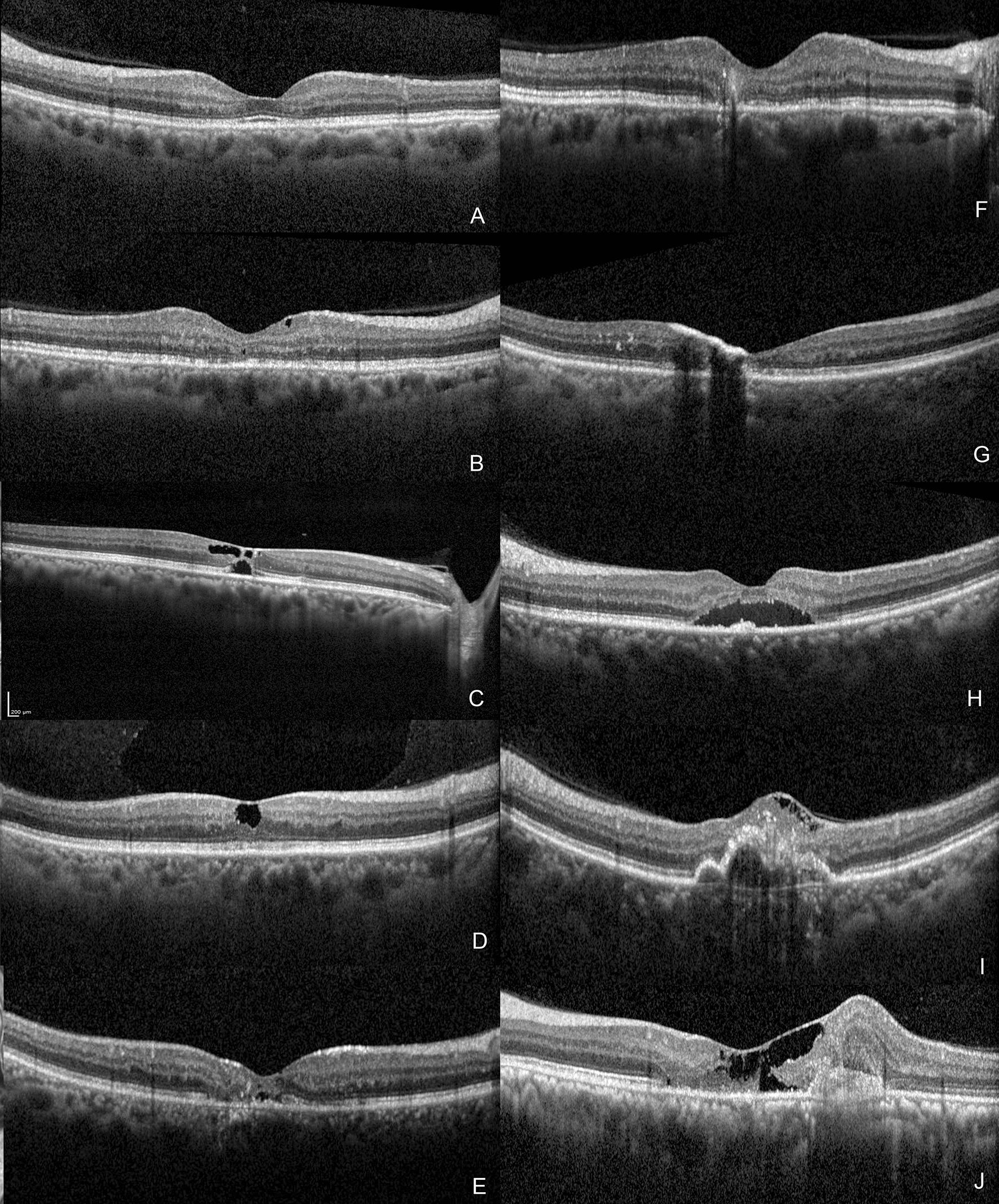
Different optical coherence tomography imaging findings seen in macular telangiectasia type 2 (MacTel type 2): **A** Increased reflectivity of the inner retina at the middle retinal layers at the temporal parafovea and asymmetric foveal contour are present. **B** Hyperreflective middle retinal layers temporal to the fovea with hyporeflective inner retinal cavity. **C** Inner and outer retinal hyporeflective cavities are noted. **D** Hyporeflective inner retinal cavity can be found at the foveal centre with an overlying internal limiting membrane drape is present. **E** Superficial retinal crystals are seen as hyperreflective spots in the superficial layer of the retina without any back shadowing and outward turning of the inner retinal layers is noted with shallow subfoveal subretinal fluid. **F** Outward turning of the inner retinal layers is noted. **G** Pigment migration to the inner retinal layers with back shadowing is noted. **H** Hyperreflective middle retinal layers with subfoveal SRF is noted. **I** Internal limiting membrane drape with foveal contour irregularity is noted. There is a hyperreflective material noted in the retinal layers breaching the retinal pigment epithelium suggestive of retino-choroidal anastomosis. **J** Foveal contour distortion with presence of internal limiting membrane drape with outward turning of the middle retinal layers and presence of subretinal neovascular membrane is noted

All eyes obtained SDOCT scans using the spectral domain Spectralis machine (Spectralis, Heidelberg Engineering, Heidelberg, Germany). 512 A-scans per line with a 30° scanning area and 25-line horizontal raster volume scans centered on the fovea were used for macular volumetric assessments. Additionally, a foveal-centered 12-line radial scan was analyzed for each eye. SDOCT scans with a quality score of > 20 were utilized for analysis and interpretation of the results. All images encompassing the macula were analyzed by independent observers from the respective centres (NR and ISA), who identified the following characteristics from inner to outer retina (Fig. 1): (1) irregularities of the foveal contour, (2) internal limiting membrane (ILM) drape sign, (3) the hyperreflectivity of the middle retinal layers (MRL), between the inner and outer plexiform layers, (4) identification of the superficial retinal crystals (5) hypo reflective inner retinal cavities, (6) hypo reflective outer retinal cavities, (7) outward bending of the inner retinal layers, (8) retinal pigment clumps (RPC) with underlying shadowing, (9) subfoveal neurosensory detachment, (10) Pseudohole, full-thickness or lamellar macular hole, (11) SRNVM, and (12) retino-choroidal anastomosis. Identification of an oval, fusiform hyperreflective lesion in the subretinal space above the retinal pigment epithelium with or without associated SRF or exudation was defined as SRNVM. Extension of the retinal neovascularisation directly to the underlying choroid following a pigment epithelial detachment and breach was defined as retino-choroidal anastomosis (RCA). Our previous publication provides a comprehensive description of these various SDOCT imaging features [7].

### Statistical tests

All data were analysed using GraphPad Prism version 9.5.1 (733) for Windows, GraphPad Software, San Diego, California USA, http://www.graphpad.com. The Shapiro-Wilk normality test was used to test the normality of the data sets. Snellen’s vision data was converted to logarithm of minimum angle of resolution (logMAR) vision for statistical analysis. Different SDOCT imaging features were described as numbers and percentages. Quantitative variables between the two groups were analysed using the Mann-Whitney U test for non-parametric data. Chi-square test was used to compare the categorical data between the two groups. Correlations between the different SDOCT biomarkers and visual acuity were analysed using the Spearman’s correlation test. The binary responses of the various SDOCT imaging features were converted to numerical values (0 = absent and 1 = present) for the purpose of studying correlation with visual acuity. Multiple variable linear regression analysis was performed between the visual acuity as the dependent variable and statistically significant SDOCT features on corelation matrix as independent variables. p values < 0.05 were considered statistically significant.

## Results

The electronic medical record system identified a total of 1,806 type 2 MacTel patients. At the time of their type 2 MacTel diagnosis, 62 eyes of 32 (2%) of these MacTel cases were younger than 40 years of age. 90% (n = 56) of the eyes examined had visual complaints, such as blurred or wavy vision. The median and the mean age was 39 and 38.44 ± 1.795 years respectively. The youngest patient in the study was 34 years. 84% (n = 27) of the patients were female, and 50% (n = 16) were diabetic. In 30 (94%) of 32 young patients with type 2 MacTel, bilateral involvement was observed. Clinical and SDOCT data was available for 62 of 32 patients’ eyes. At baseline, the median logMAR visual acuity was 0.18 (Snellen equivalent—20/30).

Table 1 depicts the clinical findings observed on retinal examination of these type 2 MacTel eyes. In 56 (90%) of the 62 eyes analyzed in the study, peri foveal greying was the most prevalent retinal finding.

**Table 1:** Clinical features noted in young patients with type 2 MacTel on retinal examination

Total no. of eyes with type 2 MacTel (n)	62
Perifoveal greying (n, %)	56 (90)
Superficial retinal crystals (n, %)	2 (3)
Right angled vessels (n, %)	8 (13)
Retinal pigment plaques (n, %)	10 (16)
SRNVM (n, %)	6 (10)

*SRNVM* subretinal neovascular membrane

Table 2 outlines the various SDOCT imaging characteristics of study participants. In 47 (76%) of 62 eyes, hyperreflective perifoveal MRL was the most prominent imaging finding detected by SDOCT. This was followed by the presence of an irregular foveal contour (74%), hyporeflective inner (35%) and outer (29%) retinal cavities, an outwardly bent IRL (24%), an ILM drape sign (21%), and a hyperreflective RPC (13%). In six (10%) eyes, subfoveal fluid and associated SRNVM were present. No full-thickness macular hole was ever observed. The differences in visual acuity in the presence or absence of these SDOCT findings are outlined in Table 3. Compared to the other SDOCT imaging characteristics, the presence of outward bending of the inner retinal layers (p = 0.047), RPC (p = 0.007), subfoveal neurosensory detachment (p = 0.048), and SRNVM (p = 0.001) significantly affected visual acuity. Correlations between the SDOCT imaging features and logMAR visual acuity was studied using the Spearman’s correlation test (Table 4). Poor vision was noted with the presence of outward turning of the inner retinal layers (r = 0.299; p = 0.018), subfoveal neurosensory detachment (r = 0.265; p = 0.037) hyperreflective RPC (r = 0.352; p = 0.005) and SRNVM (r = 0.40; p = 0.001). Multiple linear regression analysis was performed to identify the SDOCT features showing the best correlations with the visual acuity. Presence of SRNVM (p = 0.013) was the only SDOCT finding which showed statistically significant change with vision.

**Table 2:** OCT features noted in young patients with type 2 MacTel

OCT finding	Value
Foveal irregularity (n, %)	46 (74)
ILM drape sign (n, %)	13 (21)
Hyperreflective middle retinal layers (n, %)	47 (76)
Superficial retinal crystals (n, %)	6 (10)
Hyporeflective inner retinal cavities (n, %)	22 (35)
Outward bending of inner retinal layers (n, %)	15 (24)
Hypo reflective outer retinal cavities (n, %)	18 (29)
Retinal pigment clumps (n, %)	8 (13)
Sub foveal neurosensory detachment (n, %)	6 (10)
SRNVM (n, %)	6 (10)
RCA (n, %)	0 (0)

*MacTel* macular telangiectasia, *OCT* optical coherence tomography, *ILM* internal limiting membrane, *SRNVM* sub retinal neovascular membrane, *RCA* retinochoroidal anastomosis

**Table 3:** Visual acuity differences in the presence or absence of OCT imaging features

OCT finding	LogMAR visual acuity when OCT finding is present (mean ± SD)	LogMAR visual acuity when OCT finding is absent (mean ± SD)	p value
Foveal irregularity	0.248 ± 0.244	0.231 ± 0.187	0.996
ILM drape sign	0.206 ± 0.239	0.252 ± 0.228	0.527
Hyperreflective middle retinal layers	0.227 ± 0.243	0.295 ± 0.176	0.182
Superficial retinal crystals	0.227 ± 0.139	0.246 ± 0.237	0.967
Hyporeflective inner retinal cavities	0.187 ± 0.24	0.272 ± 0.22	0.108
Outward bending of inner retinal layers	0.349 ± 0.258	0.208 ± 0.209	0.047
Hypo reflective outer retinal cavities	0.277 ± 0.209	0.229 ± 0.237	0.406
Retinal pigment clumps	0.463 ± 0.239	0.21 ± 0.209	0.007
Sub foveal neurosensory detachment	0.433 ± 0.258	0.223 ± 0.217	0.048
SRNVM	0.55 ± 0.164	0.21 ± 0.209	0.001

*OCT* optical coherence tomography, *ILM* internal limiting membrane, *SRNVM* sub retinal neovascular membrane, *logMAR* logarithm of minimum angle of resolution

**Table 4:** Correlation between the different SDOCT imaging features and visual acuity (logMAR) using the Spearman’s correlation test

LogMAR visual acuity vs. OCT finding	r value	95% confidence interval	p value
Foveal irregularity	− 0.018	− 0.2733 to 0.2402	0.891
ILM drape sign	− 0.078	− 0.3286 to 0.1821	0.545
Hyperreflective middle retinal layers	− 0.142	− 0.3845 to 0.1196	0.272
Superficial retinal crystals	− 0.017	− 0.2726 to 0.2409	0.896
Hyporeflective inner retinal cavities	− 0.232	− 0.4610 to 0.0268	0.070
Outward bending of inner retinal layers	0.299	0.0459 to 0.5164	0.018
Hypo reflective outer retinal cavities	0.092	− 0.1684 to 0.3412	0.475
Retinal pigment clumps	0.352	0.1050 to 0.5586	0.005
Sub foveal neurosensory detachment	0.265	0.0091 to 0.4888	0.037
SRNVM	0.400	0.1590 to 0.5953	0.001

*OCT* optical coherence tomography, *ILM* internal limiting membrane,  *SRNVM* sub retinal neovascular membrane, *logMAR* logarithm of minimum angle of resolution, *r* Spearman’s coefficient

**Fig. 2:**
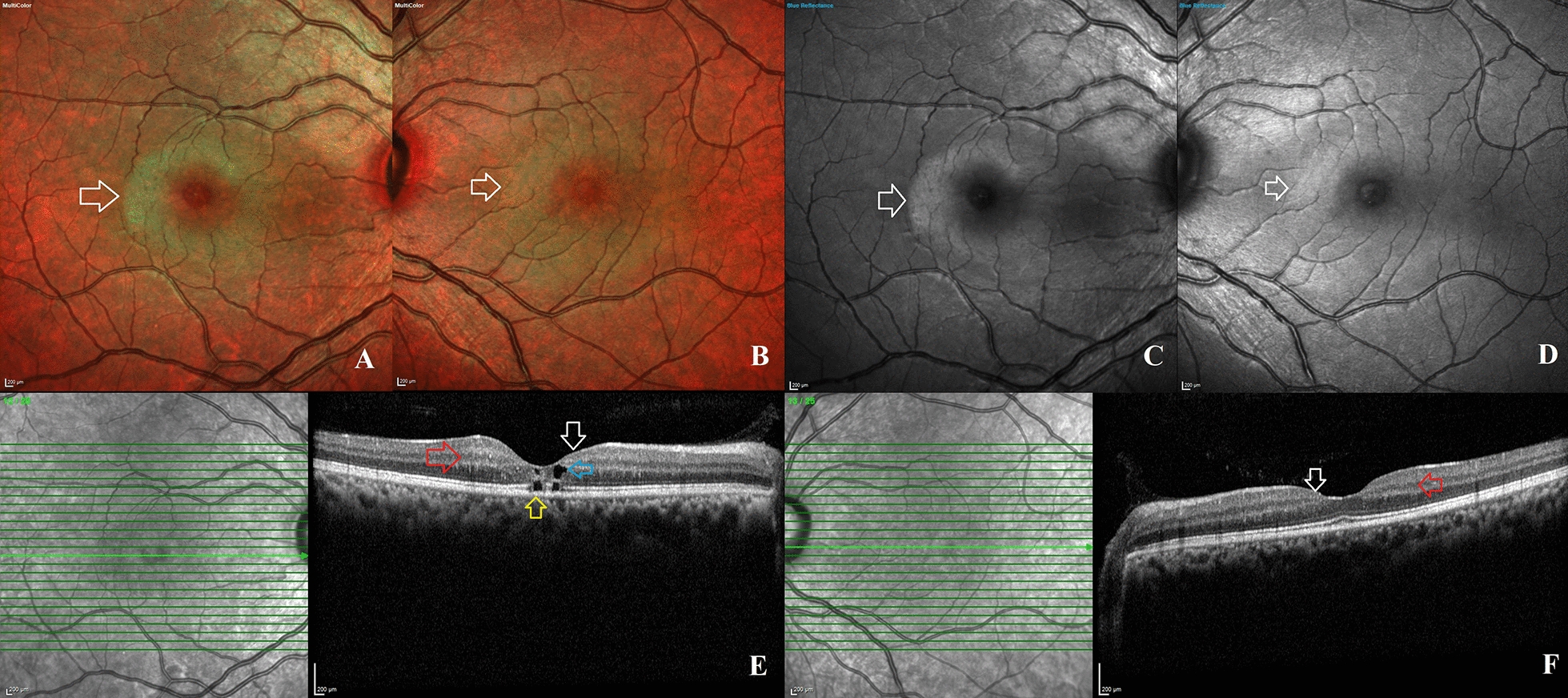
Clinical and imaging findings in a young patient presenting with bilateral non-proliferative type 2 macular telangiectasia (MacTel): These images belong to a 36-year-old diabetic female who presented to the retina clinic with blurred vision and visual acuity of 20/30 in both eyes. **A**, **B** Color fundus photographs of the right and left eye obtained with the Multicolour® imaging technology on the Spectralis machine (Heidelberg Engineering, Germany) demonstrating the typical perifoveal greying and loss of retinal transparency (white arrows) indicative of stage 2 non-proliferative disease. **C**, **D** Confocal blue reflectance imaging reveals perifoveal hyperreflectance (white arrows) in both eyes, confirming the diagnosis of type 2 MacTel disease. **E** Horizontal raster optical coherence tomography (OCT) imaging of the right eye passing through the center of the fovea reveals splaying of the foveal contour (white arrow), perifoveal hyperreflective middle retinal layers (red arrow), and inner (blue arrow) and outer retinal (yellow arrow) cavitations. **F** Horizontal raster OCT imaging of the left eye through the fovea demonstrates foveal splaying (white arrow) and perifoveal hyperreflective middle retinal layers (red arrow)

**Fig. 3:**
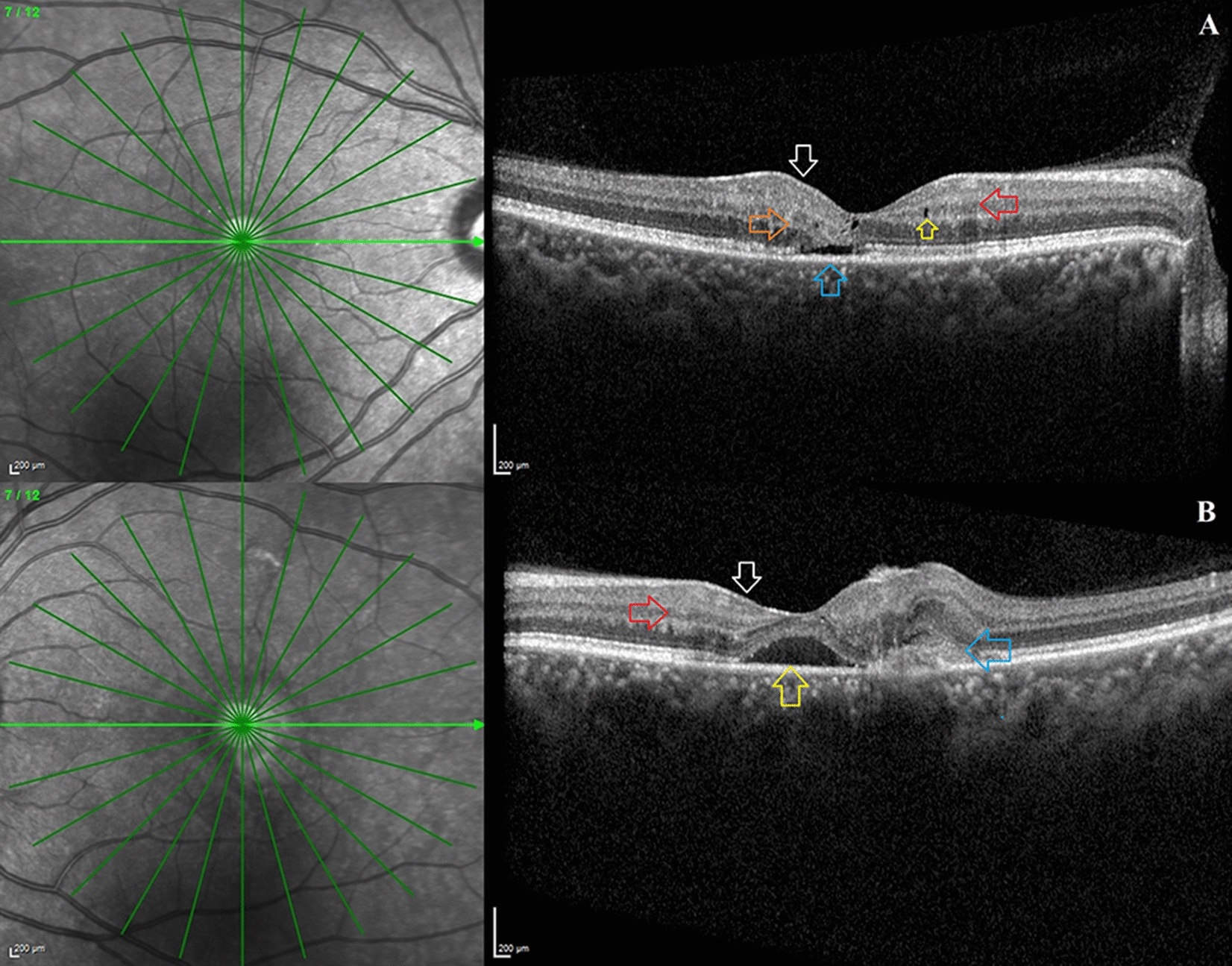
Clinical and imaging findings in a young patient presenting with proliferative type 2 macular telangiectasia (MacTel): The optical coherence tomography (OCT) images shown below are from a 39-year-old female who initially presented to the retina clinic with decreased vision in her left eye. Her presenting visual acuity in both eyes was 20/40 and 20/80, respectively. **A** A horizontal line radial OCT image of the right eye shows splaying of the foveal contour (white arrow), perifoveal hyperreflective middle retinal layers (red arrow), hyporeflective inner retinal cavities (yellow arrow), outward bending of the inner retinal layers (orange arrow), and the presence of a subfoveal neurosensory detachment in the absence of a subretinal neovascular membrane (blue arrow). **B** A horizontal line radial OCT image of the left eye shows the foveal contour splaying (white arrow) with hyperreflective middle retinal layers (red arrow) seen on the nasal aspect of the perifoveal region, as well as a frank subretinal neovascular membrane (blue arrow) and subfoveal neurosensory detachment, indicating a proliferative stage of MacTel in the left eye

For a better understanding of the SDOCT imaging characteristics, the eyes were divided into two groups: Group 1: non-proliferative type 2 MacTel (no SRNVM) and Group 2: proliferative type 2 Mactel (with SRNVM) (Figs. 2 and 3). The visual acuity of eyes in the proliferative type 2 MacTel group (logMAR VA—0.55, Snellen equivalent—20/71) was considerably worse than in the non-proliferative type 2 MacTel group (logMAR VA—0.21, Snellen equivalent—20/32). Except for the presence of RPC, all SDOCT imaging characteristics did not differ significantly between the two groups. In the proliferative type 2 MacTel group (n = 4, 67%), RPCs were found more frequently (Table 5).

**Table 5:** Comparison of the logMAR visual acuity and SDOCT imaging features between the non-proliferative and proliferative type 2 MacTel eyes

	Non-proliferative type 2 MacTel (n = 56)	Proliferative type 2 MacTel (n = 6)	p value
Mean logMAR visual acuity (Snellen equivalent)	0.21 ± 0.209 (20/32)	0.55 ± 0.164 (20/71)	0.001
Foveal irregularity (n, %)	40 (71)	6 (100)	0.325
ILM drape sign (n, %)	12 (21)	1 (17)	> 0.999
Hyperreflective middle retinal layers (n, %)	42 (75)	5 (83)	> 0.999
Superficial retinal crystals (n, %)	4 (7)	1 (17)	0.41
Hyporeflective inner retinal cavities (n, %)	19 (34)	3 (50)	0.657
Outward bending of inner retinal layers (n, %)	13 (23)	2 (33)	0.626
Hypo reflective outer retinal cavities (n, %)	18 (32)	0 (0)	0.168
Retinal pigment clumps (n, %)	4 (7)	4 (67)	0.002
Sub foveal neurosensory detachment (n, %)	4 (7)	2 (33)	0.099

*SDOCT* spectral domain optical coherence tomography, *ILM* internal limiting membrane, *logMAR* logarithm of minimum angle of resolution

## Discussion

In summary, the study revealed a significant predominance of females, with perifoveal hyperreflective MRL being the most frequently observed finding in OCT imaging. Additionally, the study identified hyperreflective RPC in the proliferative disease stage as the most relevant observation. Furthermore, it was observed that individuals with impaired visual acuity exhibited specific characteristics on SDOCT, such as the outward bending of inner retinal layers, RPC, subfoveal neurosensory detachment, and SRNVM. The current literature lacks a comprehensive description of the prevalence, clinical manifestations, and imaging characteristics of young individuals with type 2 MacTel.

Type 2 MacTel is primarily a neurodegenerative disorder affecting the perifoveal region of the macula, characterized by microvascular alterations involving the deep retinal capillary plexus predominately [1]. The etiological pattern that gives rise to type 2 MacTel is triggered by the degeneration of Müller cells. The persistent reduction of Müller cells leads to diverse alterations in the structure of the retina, encompassing changes in glial, neuronal, and retinal pigment epithelium components. Additionally, this process is associated with secondary remodeling of retinal blood vessels and the development of abnormal SRNVM [8].

In its earliest stage, type 2 MacTel is typically asymptomatic, and it is frequently not diagnosed until patients are in their fifth to eighth decades of life and present with visual symptoms. Few population-based prevalence studies have been published in the scientific literature. The prevalence rate in the Melbourne Collaborative Cohort Study in Australia ranges from 0.0045 to 0.022%, while the prevalence rate in the American Beaver Dam Eye Study is estimated to be 0.1% [2, 9]. The estimated prevalence in Nigeria was 0.06% [10]. All prevalence studies were conducted on the basis of fundus photographic gradings. The prevalence data recorded in this study is not comparable to the population-based studies described above. The current research did not provide an account of the frequency of type 2 MacTel cases observed in the hospital within the specified duration of the study. The present study aims to clarify the frequency of type 2 MacTel cases observed in patients under the age of 40, relative to the total number of diagnosed type 2 MacTel cases encountered at the hospital. The prevalence rate observed in the present study was 1.77%. There is a lack of comparative studies available to assess the prevalence rate of young individuals with type 2 MacTel in relation to our study. The present investigation suggests that the increased identification of type 2 MacTel in younger individuals could be attributed to the utilization of advanced retinal imaging techniques, including confocal blue reflectance, SDOCT, and fundus fluorescein angiography, together with conventional color fundus photographic imaging.

About 50% of the young patients with type 2 MacTel in our study had diabetes mellitus. Even in our previous publications, we had observed a high prevalence of diabetes in patients with type 2 MacTel [7, 11]. The role of Müller cells in type 2 MacTel and diabetic patients with or without diabetic retinopathy is the link between these two entities [12, 13].

In our study cohort of young individuals with type 2 MacTel, the most frequently observed characteristics were loss of retinal transparency and the presence of perifoveal greying during clinical examination. Additionally, the presence of hyperreflective MRL, primarily in the temporal perifoveal region, was a common finding on SDOCT. These observations align with the findings reported in our previous publication on SDOCT findings in older individuals with type 2 MacTel [7]. The initial manifestation of type 2 MacTel is characterized by the degeneration of Müller cells located in the perifoveal region, leading to the subsequent depletion of macular pigments, specifically lutein and zeaxanthin [14–16]. Müller cells contribute to the structural integrity of the fovea and are an integral component of the inner blood retinal barrier [17, 18]. As a result of the loss of perifoveal Müller cells in type 2 MacTel, the deep retinal capillary plexus becomes visible as hyperreflective lesions in the MRL on SDOCT. The hyperreflective MRL in type 2 MacTel must be distinguished from two clinical entities: paracentral acute middle maculopathy (PAMM) and disorganisation of the retinal inner layers (DRIL) at the macula. On SDOCT, PAMM is characterized by paracentral retinal hyperreflectivity in the middle retinal layers with underlying outer retinal layer shadowing, and it occurs in a patient who has recently complained of central scotoma or blurred vision [19]. In type 2 MacTel, there is no shadowing of the outer retinal layer and the symptoms are not acute. The retinal layers stratification in the MRL appears to be maintained in type 2 MacTel. This must be distinguished from DRIL, in which the inner retinal layer stratification is lost [20].

90% (n = 56) of the eyes in the current study cohort were diagnosed with a non-proliferative stage of type 2 MacTel. These eyes presented with significantly better visual acuity than those with proliferative type 2 MacTel. In this study, the presence of RPC coincided with a proliferative stage of type 2 MacTel (7% vs. 67%, p = 0.002), which is an important finding. Leung et al. made comparable observations in their study and proposed modifying the current classification of type 2 MacTel based on pigment plaque characteristics [21]. Furthermore, our previous research publication pertaining to the attributes of RPCs in type 2 MacTel has identified additional characteristics. These include the prevalence of RPCs in females, the existence of larger pigment sizes, and the localization of RPCs above the outer plexiform layer. These identified characteristics have demonstrated a correlation with impaired vision and the manifestation of a proliferative disease [22].

Further differentiation of the SDOCT findings between those affecting the inner retinal layers and those affecting the outer retinal layers revealed that in our cohort of young type 2 MacTel patients, changes that predominantly involved the outer retinal layers were associated with significantly worse vision. Similar observations were made in our previous publication on SDOCT imaging characteristics in type 2 MacTel and their correlation with visual acuity [7]. Regardless of the age at which type 2 MacTel manifests, the involvement of the outer retinal layers is indicative of a poor visual prognosis.

There are several limitations related to our study. One significant constraint of this retrospective study is the lack of a control group consisting of individuals aged 40 years and above who have type 2 MacTel. Furthermore, the study solely provided a description of the SDOCT imaging outcomes during the initial presentation, without any reference to subsequent follow-up examinations. This study found a significant correlation between structural changes on SDOCT and visual acuity, with no other functional outcomes showing a similar association. To enhance the depth of the analysis, it would have been advantageous to incorporate additional important functional measures, including near vision, microperimetry changes, and multifocal electroretinography changes, in conjunction with the observed SDOCT changes. The investigation did not include an examination and correlation of additional outer retinal SDOCT observations, such as the discontinuity of the ELM, ellipsoid zone, and interdigitation zone, with visual acuity. Accurate identification of these findings on SDOCT, particularly during the proliferative stages of the disease, posed a significant challenge. The classification of SDOCT features based on their location and relationship to the fovea was not conducted in the study.

Nevertheless, it is our belief that despite these constraints, our research brings attention to the frequency, clinical manifestations, and imaging characteristics of a specific group of individuals with type 2 MacTel who are below the age of 40 at the time of diagnosis. Future prospective studies are planned to investigate the symmetry of the disease, make comparisons with previous cases of type 2 MacTel, and examine longitudinal changes in SDOCT over a period of time.

To summarize, type 2 MacTel is infrequent in individuals below the age of 40, often affecting both eyes, strongly linked to diabetes mellitus, and primarily impacts visual function during the later stages of the condition.

## Data Availability

The datasets used and/or analysed during the current study are available from the corresponding author on reasonable request.

## Abbreviations

MacTel: Macular telangiectasia
SRNVM: Subretinal neovascular membrane
LogMAR: Logarithm of minimum angle of resolution
SDOCT: Spectral domain optical coherence tomography
ILM: Internal limiting membrane
MRL: Middle retinal layers
RPC: Retinal pigment clumps

## Declarations

### Animal research

“This article does not contain any studies with animals performed by any of the authors.”
